# Draft Genome Sequence of *Brevundimonas* sp. Strain SH203, Producing Cellouronate (β-1,4-Linked Polyglucuronate) Lyase

**DOI:** 10.1128/genomeA.00262-17

**Published:** 2017-05-04

**Authors:** Tomohiro Suzuki, Masako Kikuchi, Naotake Konno, Naoto Habu

**Affiliations:** a Center for Bioscience Research and Education, Utsunomiya University, Utsunomiya, Tochigi, Japan; b Department of Applied Biological Chemistry, School of Agriculture, Utsunomiya University, Utsunomiya, Tochigi, Japan

## Abstract

In this study, we report the draft genome sequence of *Brevundimonas* sp. strain SH203, which was previously isolated from natural soil and has the ability to degrade β-1,4-polygluculonate (cellouronate). This genomic information may provide new insight into the mechanisms by which cellouronate is degraded.

## GENOME ANNOUNCEMENT

A bacterial strain with the ability to degrade cellouronate and isolated from natural soil was identified as *Brevundimonas* sp. strain SH203 by comparing its 16S rRNA gene sequence with that in the GenBank database (1). *Brevundimonas* sp. SH203 was reported to degrade β-1,4-polygluculonate (cellouronate), which is artificially prepared from regenerated cellulose by TEMPO (2,2,6,6-tetramethylpiperidine-1-oxyl radical)-mediated oxidation. We have purified two types of cellouronate lyases from the strain, CUL-I and CUL-II, which catalyze the depolymerization of cellouronate by cleaving glycoside bonds through β-elimination (1–4).

*Brevundimonas* sp. SH203 was cultivated in 10 ml of LB broth at 30°C for 2 days, and genomic DNA extracted using the DNeasy blood and tissue kit, followed by RNase A digestion. The sheared genomic DNA, resulting in fragments with an average size of 550 bp, and a paired-end DNA library were constructed using the TruSeq DNA PCR-free library preparation kit, according to the manufacturer’s instructions. The DNA library then sequenced on a MiSeq instrument at the Center for Bioscience Research and Education, Utsunomiya University, Japan. The raw sequencing data (2 × 301 bp in length) were trimmed to remove low-quality ends (<15) and adapters, using Trimmomatic (version 0.36) (5). To remove contaminant sequences or sequence errors, reads with low coverage (<5×) were removed using khmer (version 2.0) (6), resulting in 1,084,068 high-quality reads (542,034 pairs) totaling 266 Mb (~84-fold coverage). The high-quality reads were further assembled using SPAdes version 3.9.0 (7), with the “careful” option selected, and contigs of less than 200 bp were eliminated. The draft genome of *Brevundimonas* sp. SH203 assembled into 16 contigs, with a total length of 3,145,540 bp (*N*_50_, 497,772 bp) and 67.6% G+C content.

The resultant draft genome sequence was annotated using Prokka version 1.11 (8). Furthermore, tRNA genes and rRNA sequences were predicted using tRNAscan-SE version 1.3.1 (9) and RNAmer version 1.2 (10), respectively. The annotated genome contains 2,961 protein-coding sequences, 47 tRNA genes, and 3 rRNA sequences. Functional annotation of the predicted proteins was conducted using Clusters of Orthologous Groups (COG) (11) and Pfam (12). Among 2,961 proteins, 2,496 (84.2%) and 2,469 (83.3%) proteins were annotated by COG and Pfam, respectively. From the results of Pfam analysis, one alginate-lyase (PF05426) and three β-eliminating lyase (PF01212) domain-containing sequences were detected which might be involved in cellouronate degradation. This genomic information may provide new insight into the mechanisms by which cellouronate is degraded.

### Accession number(s).

The draft genome sequence of *Brevundimonas* sp. strain SH203 been deposited to the DDBJ/EMBL/GenBank database under the accession no. BDMM00000000.

## References

[1] Konno N, Habu N, Maeda I, Azuma N, Isogai A 2006 Cellouronate (β-1,4-linked polyglucuronate) lyase from *Brevundimonas* sp. SH203: purification and characterization. Carbohydr Polym 64:589–596. doi:10.1016/j.carbpol.2005.11.015.

[2] Konno N, Habu N, Iihashi N, Isogai A 2008 Purification and characterization of exo-type cellouronate lyase. Cellulose 15:453–463. doi:10.1007/s10570-007-9195-z.

[3] Konno N, Igarashi K, Habu N, Samejima M, Isogai A 2009 Cloning of the *Trichoderma reesei* cDNA encoding a glucuronan lyase belonging to a novel polysaccharide lyase family. Appl Environ Microbiol 75:101–107. doi:10.1128/AEM.01749-08. 18978091 PMC2612218

[4] Konno N, Ishida T, Igarashi K, Fushinobu S, Habu N, Samejima M, Isogai A 2009 Crystal structure of polysaccharide lyase family 20 endo-β-1,4-glucuronan lyase from the filamentous fungus *Trichoderma reesei*. FEBS Lett 583:1323–1326. doi:10.1016/j.febslet.2009.03.034. 19306878

[5] Bolger AM, Lohse M, Usadel B 2014 Trimmomatic: a flexible trimmer for Illumina sequence data. Bioinformatics 30:2114–2120. doi:10.1093/bioinformatics/btu170. 24695404 PMC4103590

[6] Crusoe MR, Alameldin HF, Awad S, Boucher E, Caldwell A, Cartwright R, Charbonneau A, Constantinides B, Edvenson G, Fay S, Fenton J, Fenzl T, Fish J, Garcia-Gutierrez L, Garland P, Gluck J, Gonzalez I, Guermond S, Guo J, Gupta A, Herr JR, Howe A, Hyer A, Harpfer A, Irber L, Kidd R, Lin D, Lippi J, Mansour T, McA’Nulty P, McDonald E, Mizzi J, Murray KD, Nahum JR, Nanlohy K, Nederbragt AJ, Ortiz-Zuazaga H, Ory J, Pell J, Pepe-Ranney C, Russ ZN, Schwarz E, Scott C, Seaman J, Sievert S, Simpson J, Skennerton CT, Spencer J, Srinivasan R, Standage D, et al. 2015 The Khmer software package: enabling efficient nucleotide sequence analysis. F1000Res 4:900. 26535114 10.12688/f1000research.6924.1 PMC4608353

[7] Bankevich A, Nurk S, Antipov D, Gurevich AA, Dvorkin M, Kulikov AS, Lesin VM, Nikolenko SI, Pham S, Prjibelski AD, Pyshkin AV, Sirotkin AV, Vyahhi N, Tesler G, Alekseyev MA, Pevzner PA 2012 SPAdes: a new genome assembly algorithm and its applications to single-cell sequencing. J Comput Biol 19:455–477. doi:10.1089/cmb.2012.0021. 22506599 PMC3342519

[8] Seemann T 2014 Prokka: rapid prokaryotic genome annotation. Bioinformatics 30:2068–2069. doi:10.1093/bioinformatics/btu153. 24642063

[9] Schattner P, Brooks AN, Lowe TM 2005 The tRNAscan-SE, snoscan and snoGPS Web servers for the detection of tRNAs and snoRNAs. Nucleic Acids Res 33:W686–W689. doi:10.1093/nar/gki366. 15980563 PMC1160127

[10] Lagesen K, Hallin P, Rødland EA, Staerfeldt H-H, Rognes T, Ussery DW 2007 RNAmmer: consistent and rapid annotation of ribosomal RNA genes. Nucleic Acids Res 35:3100–3108. doi:10.1093/nar/gkm160. 17452365 PMC1888812

[11] Galperin MY, Makarova KS, Wolf YI, Koonin EV 2015 Expanded microbial genome coverage and improved protein family annotation in the COG database. Nucleic Acids Res 43:D261–D269. doi:10.1093/nar/gku1223. 25428365 PMC4383993

[12] Finn RD, Bateman A, Clements J, Coggill P, Eberhardt RY, Eddy SR, Heger A, Hetherington K, Holm L, Mistry J, Sonnhammer ELL, Tate J, Punta M 2014 Pfam: the protein families database. Nucl Acids Res 42:D222–D230. doi:10.1093/nar/gkt1223. 24288371 PMC3965110

